# 1,3-Bis[(4-methylbenzylidene)amino­oxy]propane

**DOI:** 10.1107/S1600536809042147

**Published:** 2009-10-17

**Authors:** Jian-Chao Wu, Su-Xia Gao, Wen-Kui Dong, Jun-Feng Tong, Li Li

**Affiliations:** a School of Chemical and Biological Engineering, Lanzhou Jiaotong University, Lanzhou 730070, People’s Republic of China; b School of Environmental Science and Municipal Engineering, Lanzhou Jiaotong University, Lanzhou 730070, People’s Republic of China

## Abstract

The title bis­oxime compound, C_19_H_22_N_2_O_2_, synthesized by the reaction of 4-methyl-2-hydroxy­benzaldehyde with 1,3-bis­(amino­oxy)propane in ethanol, adopts a V-shaped conformation. The dihedral angle between the rings is 84.59 (3)°. The mol­ecule is disposed about a crystallographic twofold rotation axis, with one C atom lying on the axis. In the crystal, mol­ecules are packed by C—H⋯π(Ph) inter­actions, forming chains.

## Related literature

For bis­oximes and their applications, see: Akine *et al.* (2005 ▶); Atwood & Harvey (2001 ▶); Dong *et al.* (2008 ▶, 2009 ▶); He *et al.* (2008 ▶); Yeap *et al.* (2008 ▶). 
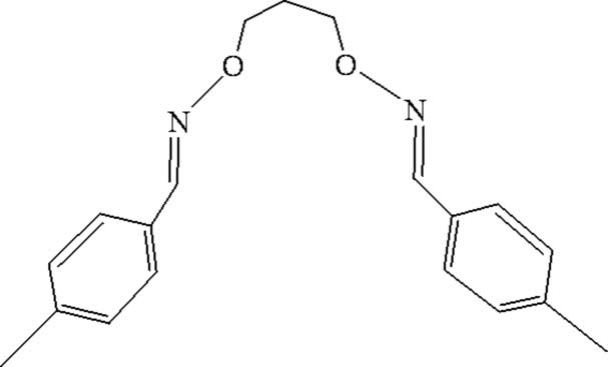


## Experimental

### 

#### Crystal data

C_19_H_22_N_2_O_2_
*M* *_r_* = 310.39
Monoclinic, 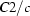

*a* = 29.843 (2) Å
*b* = 4.8668 (7) Å
*c* = 12.1202 (11) Å
β = 98.568 (1)°
*V* = 1740.7 (3) Å^3^
*Z* = 4
Mo *K*α radiation
μ = 0.08 mm^−1^
*T* = 298 K
0.43 × 0.13 × 0.07 mm

#### Data collection

Siemens SMART CCD area-detector diffractometer
Absorption correction: multi-scan (*SADABS*; Sheldrick, 1996 ▶) *T*_min_ = 0.968, *T*_max_ = 0.995
4227 measured reflections
1530 independent reflections
831 reflections with *I* > 2σ(*I*)
*R*_int_ = 0.062

#### Refinement

*R*[*F* ^2^ > 2σ(*F* ^2^)] = 0.058
*wR*(*F* ^2^) = 0.175
*S* = 1.12
1530 reflections
106 parameters
H-atom parameters constrained
Δρ_max_ = 0.13 e Å^−3^
Δρ_min_ = −0.20 e Å^−3^

### 

Data collection: *SMART* (Siemens, 1996 ▶); cell refinement: *SAINT* (Siemens, 1996 ▶); data reduction: *SHELXTL* (Sheldrick, 2008 ▶); program(s) used to solve structure: *SHELXS97* (Sheldrick, 2008 ▶); program(s) used to refine structure: *SHELXL97* (Sheldrick, 2008 ▶); molecular graphics: *SHELXTL*; software used to prepare material for publication: *SHELXTL*.

## Supplementary Material

Crystal structure: contains datablocks global, I. DOI: 10.1107/S1600536809042147/hg2578sup1.cif

Structure factors: contains datablocks I. DOI: 10.1107/S1600536809042147/hg2578Isup2.hkl

Additional supplementary materials:  crystallographic information; 3D view; checkCIF report

## Figures and Tables

**Table 1:** Hydrogen-bond geometry (Å, °)

*D*—H⋯*A*	*D*—H	H⋯*A*	*D*⋯*A*	*D*—H⋯*A*
C10—H10*C*⋯*Cg*1	0.96	2.73	3.614 (2)	153

*Cg*1 is the centroid of the C4–C9 ring.

**Fig. 1.**
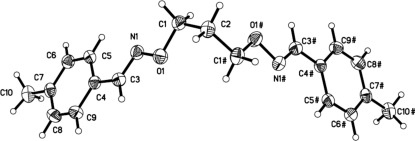
The molecule structure of the title compound with atom numbering [Symmetry codes: -x, y, 1/2 - z]. Displacement ellipsoids for non-hydrogen atoms are drawn at the 30% probability level.

**Fig. 2.**
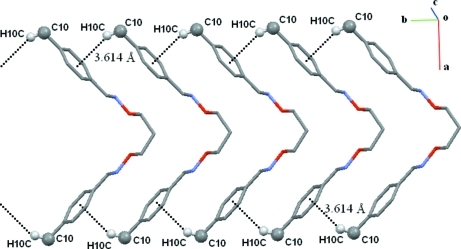
Part of the supramolecular structure of the title compound. C—H···π(Ph) interactions are shown as dashed lines.

## supplementary crystallographic information

### Comment

Much interest has been focused on bisoxime compounds, in which the large electronegativity of O atoms is expected to affect strongly the electronic properties of the nitrogen atoms, and exhibit high stability against imine metathesis reactions (Akine *et al.*, 2005). Some of them or their metal complexes are used in wide field due to their variety of applications, especially for catalysis and biological processes, magnetism, and supramolecular architectures (Atwood *et al.*, 2001; Yeap *et al.*, 2008; Dong *et al.*, 2008). Herein, the synthesis and structure of 4,4'-dimethyl-1,3-[propenedioxybis(nitrilomethylidyne)]dibenzene (I) is reported (Fig. 1).

The single-crystal structure of (I) is built up by discrete C_19_H_22_N_2_O_2_ molecules, in which all bond lengths are in normal ranges. The title compound adopts a V-shaped configuration with the dihedral angle between the two halves of the molecule is 85.82 (3)°. The molecules are disposed about a crystallographic two-fold rotation axis. This structure is similar to that observed in our previously reported salen-type bisoxime compounds (He *et al.*, 2008). The packing of the molecule is controlled by C—H···π(Ph) interactions linking molecules into infinite supramolecular structure along *b* axis.

### Experimental

4,4'-Dimethyl-1,3-[propenedioxybis(nitrilomethylidyne)]dibenzene was synthesized according to an analogous method reported earlier (Dong *et al.*, 2009). To an ethanol solution (4 ml) of 4-methyl-2-hydroxybenzaldehyde (243.2 mg, 2.02 mmol) was added an ethanol solution (4 ml) of 1,3-bis(aminooxy)propane (108.3 mg, 1.02 mmol). The reaction mixture was stirred at 328–333 K for 14 h. After cool to room temperature, no precipitate was formed, which was concentrated to about 1 ml under reduced pressure. The precipitate formed was separated by filtration, and washed several times with n-hexane. The product was dried under vacuum to yield 189.4 mg of (I). Yield, 60.4%. m. p. 329–330 K. Anal. Calcd. for C_19_H_22_N_2_O_2_: C, 73.52; H, 7.14; N, 9.03. Found: C, 73.49; H, 7.01; N, 9.39.

Colorless needle-like single crystals suitable for X-ray diffraction studies were obtained after about four weeks by slow evaporation from an acetonitrile solution of (I).

### Refinement

Non-H atoms were refined anisotropically. H atoms were treated as riding atoms with distances C—H = 0.96 Å (CH_3_), 0.97 Å (CH_2_),0.93 Å (CH) and *U*_iso_(H) = 1.2 *U*_eq_(C).

### Crystal data

**Table:**

C_19_H_22_N_2_O_2_	*F*(000) = 664
*M**_r_* = 310.39	*D*_x_ = 1.184 Mg m^−^^3^
Monoclinic, *C*2/*c*	Melting point = 329–330 K
Hall symbol: -C 2yc	Mo *K*α radiation, λ = 0.71073 Å
*a* = 29.843 (2) Å	Cell parameters from 780 reflections
*b* = 4.8668 (7) Å	θ = 2.8–25.2°
*c* = 12.1202 (11) Å	µ = 0.08 mm^−^^1^
β = 98.568 (1)°	*T* = 298 K
*V* = 1740.7 (3) Å^3^	Needle-like, colorless
*Z* = 4	0.43 × 0.13 × 0.07 mm

### Data collection

**Table:**

Siemens SMART CCD area-detector diffractometer	1530 independent reflections
Radiation source: fine-focus sealed tube	831 reflections with *I* > 2σ(*I*)
graphite	*R*_int_ = 0.062
phi and ω scans	θ_max_ = 25.0°, θ_min_ = 2.8°
Absorption correction: multi-scan (*SADABS*; Sheldrick, 1996)	*h* = −34→25
*T*_min_ = 0.968, *T*_max_ = 0.995	*k* = −5→5
4227 measured reflections	*l* = −14→14

### Refinement

**Table:**

Refinement on *F*^2^	Primary atom site location: structure-invariant direct methods
Least-squares matrix: full	Secondary atom site location: difference Fourier map
*R*[*F*^2^ > 2σ(*F*^2^)] = 0.058	Hydrogen site location: inferred from neighbouring sites
*wR*(*F*^2^) = 0.175	H-atom parameters constrained
*S* = 1.12	*w* = 1/[σ^2^(*F*_o_^2^) + (0.071*P*)^2^] where *P* = (*F*_o_^2^ + 2*F*_c_^2^)/3
1530 reflections	(Δ/σ)_max_ < 0.001
106 parameters	Δρ_max_ = 0.13 e Å^−^^3^
0 restraints	Δρ_min_ = −0.20 e Å^−^^3^

### Special details

**Table:**

Geometry. All e.s.d.'s (except the e.s.d. in the dihedral angle between two l.s. planes) are estimated using the full covariance matrix. The cell e.s.d.'s are taken into account individually in the estimation of e.s.d.'s in distances, angles and torsion angles; correlations between e.s.d.'s in cell parameters are only used when they are defined by crystal symmetry. An approximate (isotropic) treatment of cell e.s.d.'s is used for estimating e.s.d.'s involving l.s. planes.
Refinement. Refinement of *F*^2^ against ALL reflections. The weighted *R*-factor *wR* and goodness of fit *S* are based on *F*^2^, conventional *R*-factors *R* are based on *F*, with *F* set to zero for negative *F*^2^. The threshold expression of *F*^2^ > σ(*F*^2^) is used only for calculating *R*-factors(gt) *etc*. and is not relevant to the choice of reflections for refinement. *R*-factors based on *F*^2^ are statistically about twice as large as those based on *F*, and *R*- factors based on ALL data will be even larger.

### Fractional atomic coordinates and isotropic or equivalent isotropic displacement parameters (Å^2^)

**Table:**

	*x*	*y*	*z*	*U*_iso_*/*U*_eq_	
N1	0.58112 (8)	0.7298 (5)	0.3832 (2)	0.0491 (7)	
O1	0.54391 (7)	0.5473 (4)	0.36901 (16)	0.0562 (7)	
C1	0.54272 (10)	0.4006 (7)	0.2661 (2)	0.0530 (9)	
H1A	0.5691	0.2826	0.2691	0.064*	
H1B	0.5426	0.5283	0.2046	0.064*	
C2	0.5000	0.2312 (9)	0.2500	0.0532 (12)	
H2	0.4999	0.1136	0.3146	0.064*	
C3	0.58513 (10)	0.8441 (7)	0.4781 (2)	0.0493 (8)	
H3	0.5646	0.7979	0.5258	0.059*	
C4	0.62026 (9)	1.0435 (6)	0.5161 (2)	0.0436 (8)	
C5	0.65245 (10)	1.1316 (7)	0.4504 (2)	0.0494 (8)	
H5	0.6518	1.0609	0.3790	0.059*	
C6	0.68507 (10)	1.3223 (7)	0.4909 (2)	0.0518 (9)	
H6	0.7060	1.3778	0.4458	0.062*	
C7	0.68736 (10)	1.4340 (6)	0.5977 (2)	0.0487 (8)	
C8	0.65537 (11)	1.3458 (7)	0.6621 (2)	0.0558 (9)	
H8	0.6561	1.4163	0.7336	0.067*	
C9	0.62262 (10)	1.1564 (6)	0.6225 (2)	0.0518 (8)	
H9	0.6016	1.1026	0.6678	0.062*	
C10	0.72298 (11)	1.6435 (7)	0.6406 (3)	0.0653 (10)	
H10A	0.7515	1.5904	0.6194	0.098*	
H10B	0.7258	1.6534	0.7204	0.098*	
H10C	0.7143	1.8201	0.6092	0.098*	

### Atomic displacement parameters (Å^2^)

**Table:**

	*U*^11^	*U*^22^	*U*^33^	*U*^12^	*U*^13^	*U*^23^
N1	0.0415 (15)	0.0518 (18)	0.0529 (15)	−0.0036 (13)	0.0040 (11)	−0.0018 (13)
O1	0.0463 (13)	0.0644 (16)	0.0579 (13)	−0.0126 (11)	0.0075 (10)	−0.0103 (12)
C1	0.0468 (19)	0.052 (2)	0.0585 (18)	0.0048 (17)	0.0032 (14)	−0.0096 (17)
C2	0.050 (3)	0.044 (3)	0.063 (3)	0.000	−0.001 (2)	0.000
C3	0.0466 (18)	0.054 (2)	0.0473 (17)	0.0002 (16)	0.0078 (13)	−0.0002 (16)
C4	0.0435 (18)	0.0414 (19)	0.0448 (16)	0.0022 (14)	0.0030 (13)	0.0016 (14)
C5	0.0487 (19)	0.058 (2)	0.0404 (15)	0.0010 (17)	0.0023 (14)	−0.0006 (15)
C6	0.0481 (19)	0.055 (2)	0.0524 (18)	0.0002 (17)	0.0069 (14)	0.0061 (16)
C7	0.053 (2)	0.0372 (19)	0.0520 (17)	0.0036 (16)	−0.0058 (15)	0.0032 (16)
C8	0.071 (2)	0.051 (2)	0.0448 (16)	0.0016 (18)	0.0070 (16)	−0.0050 (16)
C9	0.057 (2)	0.055 (2)	0.0456 (17)	−0.0020 (17)	0.0117 (14)	−0.0012 (16)
C10	0.069 (2)	0.050 (2)	0.071 (2)	−0.0028 (19)	−0.0081 (18)	0.0022 (18)

### Geometric parameters (Å, °)

**Table:**

N1—C3	1.268 (3)	C5—C6	1.381 (4)
N1—O1	1.412 (3)	C5—H5	0.9300
O1—C1	1.433 (3)	C6—C7	1.397 (4)
C1—C2	1.506 (4)	C6—H6	0.9300
C1—H1A	0.9700	C7—C8	1.388 (4)
C1—H1B	0.9700	C7—C10	1.508 (4)
C2—C1^i^	1.506 (4)	C8—C9	1.377 (4)
C2—H2	0.9700	C8—H8	0.9300
C3—C4	1.452 (4)	C9—H9	0.9300
C3—H3	0.9300	C10—H10A	0.9600
C4—C9	1.394 (4)	C10—H10B	0.9600
C4—C5	1.403 (4)	C10—H10C	0.9600
			
C3—N1—O1	110.7 (2)	C4—C5—H5	119.7
N1—O1—C1	109.6 (2)	C5—C6—C7	121.7 (3)
O1—C1—C2	107.2 (2)	C5—C6—H6	119.2
O1—C1—H1A	110.3	C7—C6—H6	119.2
C2—C1—H1A	110.3	C8—C7—C6	117.3 (3)
O1—C1—H1B	110.3	C8—C7—C10	121.6 (3)
C2—C1—H1B	110.3	C6—C7—C10	121.1 (3)
H1A—C1—H1B	108.5	C9—C8—C7	121.6 (3)
C1—C2—C1^i^	113.6 (4)	C9—C8—H8	119.2
C1—C2—H2	108.8	C7—C8—H8	119.2
C1^i^—C2—H2	108.8	C8—C9—C4	121.4 (3)
N1—C3—C4	123.2 (3)	C8—C9—H9	119.3
N1—C3—H3	118.4	C4—C9—H9	119.3
C4—C3—H3	118.4	C7—C10—H10A	109.5
C9—C4—C5	117.4 (3)	C7—C10—H10B	109.5
C9—C4—C3	119.4 (3)	H10A—C10—H10B	109.5
C5—C4—C3	123.2 (3)	C7—C10—H10C	109.5
C6—C5—C4	120.7 (3)	H10A—C10—H10C	109.5
C6—C5—H5	119.7	H10B—C10—H10C	109.5
			
C3—N1—O1—C1	−174.3 (2)	C4—C5—C6—C7	0.1 (5)
N1—O1—C1—C2	−174.5 (2)	C5—C6—C7—C8	−0.1 (4)
O1—C1—C2—C1^i^	65.14 (18)	C5—C6—C7—C10	−179.6 (3)
O1—N1—C3—C4	−179.6 (2)	C6—C7—C8—C9	−0.1 (4)
N1—C3—C4—C9	−179.1 (3)	C10—C7—C8—C9	179.4 (3)
N1—C3—C4—C5	1.2 (5)	C7—C8—C9—C4	0.3 (5)
C9—C4—C5—C6	0.2 (4)	C5—C4—C9—C8	−0.4 (4)
C3—C4—C5—C6	179.9 (3)	C3—C4—C9—C8	179.9 (3)

Symmetry codes: (i) −*x*+1, *y*, −*z*+1/2.

### Hydrogen-bond geometry (Å, °)

**Table:**

*D*—H···*A*	*D*—H	H···*A*	*D*···*A*	*D*—H···*A*
C10—H10C···Cg1	0.96	2.73	3.614 (2)	153
